# First-trimester lipoprotein(a) and longitudinal renal biomarker trajectories preceding preeclampsia: a pilot cohort study

**DOI:** 10.1007/s00404-026-08508-x

**Published:** 2026-07-02

**Authors:** María Elena Lozoya-Angulo, Francisco Gerardo Cañizares-Hernández, Sara Requena-López, José Antonio Noguera-Velasco, Javier Sánchez-Romero, Catalina de Paco-Matallana

**Affiliations:** 1 Clinical Analysis Service, University Hospital Virgen de La Arrixaca, Ctra. Madrid-Cartagena, s/n, 30120 El Palmar, Murcia Spain; 2 Biomedical Research Institute of Murcia Pascual Parrilla–IMIB, Crtra. Madrid-Cartagena s/n, 30120 El Palmar, Murcia Spain; 3 Department of Obstetrics and Gynecology, ‘Virgen de La Arrixaca’ University Hospital, Ctra. Madrid-Cartagena, s/n, 30120 El Palmar, Murcia Spain; 4 Department of Obstetrics and Gynecology, Pediatrics and Surgery, University of Murcia, Ctra. Madrid-Cartagena, s/n, 30120 El Palmar, Murcia Spain

**Keywords:** Preeclampsia, Cystatin C, Uric acid, Lipoprotein(a), Kidney, Hypertension

## Abstract

**Purpose:**

To evaluate whether early vascular susceptibility, reflected by first-trimester lipoprotein(a), together with longitudinal renal biomarker trajectories across pregnancy, is associated with the subsequent development of preeclampsia.

**Methods:**

Retrospective analysis of prospectively collected data from a high-risk pregnancy cohort. Maternal serum inflammatory (interleukin-6, C-reactive protein), lipid (lipoprotein(a), triglycerides, total cholesterol), and renal (uric acid, cystatin C) biomarkers were measured at 11–13, 19–22, and 32 weeks of gestation. Longitudinal changes were assessed using population-averaged models including trimester, preeclampsia status, and their interaction, adjusted for maternal age and body mass index. First-trimester lipoprotein(a) was evaluated using adjusted logistic regression.

**Results:**

Among 119 women, 20 developed preeclampsia. First-trimester lipoprotein(a) concentrations were higher in women who later developed preeclampsia and were independently associated with increased risk (OR 2.38; *p* < 0.001). Longitudinal analyses showed significant trimester-by-preeclampsia interactions for uric acid and cystatin C (both *p* = 0.03), indicating progressive divergence across pregnancy. Inflammatory biomarkers showed early between-group differences without significant longitudinal divergence.

**Conclusions:**

Our findings suggest that preeclampsia may be associated with both early maternal vascular susceptibility and progressive alterations in renal biomarkers during pregnancy. Elevated first-trimester lipoprotein(a) may reflect an underlying vascular phenotype, while divergent renal biomarker trajectories highlight the dynamic evolution of the disease before clinical onset, although these findings require validation in larger prospective studies.

**Supplementary Information:**

The online version contains supplementary material available at https://doi.org/10.1007/s00404-026-08508-x.

## What does this study adds to the clinical work?

**Table:** 

This study suggests that preeclampsia may be anticipated by combining a static marker of early vascular susceptibility (first-trimester lipoprotein(a)) with dynamic longitudinal assessment of renal biomarkers. Monitoring longitudinal biomarker trajectories may provide additional insight into the biological evolution of preeclampsia.	

## Introduction

Preeclampsia is a major hypertensive disorder of pregnancy and a leading cause of maternal and perinatal morbidity and mortality worldwide [1, 2]. Although its clinical manifestations typically emerge in the second half of pregnancy, preeclampsia is increasingly recognized as a complex and multifactorial disorder involving abnormal placentation, systemic inflammation, metabolic dysregulation, endothelial dysfunction, and renal impairment [3–5]. Importantly, these pathophysiological processes are dynamic and evolve throughout gestation, suggesting that the biological profile of women who develop preeclampsia changes progressively over time rather than appearing abruptly at clinical onset.

Systemic inflammation plays a central role in the pathogenesis of preeclampsia. Among inflammatory mediators, interleukin-6 (IL-6) has been implicated in endothelial activation, oxidative stress, and vascular dysfunction [6, 7]. High levels of IL-6 have been reported in women with established preeclampsia compared with normotensive pregnancies [8, 9]. However, most investigations have relied on single time-point measurements, and longitudinal data describing the evolution of IL-6 across pregnancy in women who develop preeclampsia remain limited and inconsistent.

Metabolic and lipid abnormalities represent an additional component of preeclampsia pathophysiology that remains less extensively explored. Pregnancy is characterized by physiological hyperlipidemia, particularly in the second and third trimester. However, some studies found increased triglyceride concentrations and changes in lipoprotein metabolism associated with endothelial dysfunction, oxidative stress, and placental vascular pathology [10–12]. Others have also reported higher triglyceride and total cholesterol levels in women with preeclampsia compared with unaffected pregnancies [13, 14].

Lipoprotein(a) (Lp(a)) is a genetically determined lipoprotein with pro-atherogenic and pro-inflammatory properties that has been strongly linked to endothelial dysfunction and cardiovascular disease [15, 16]. Several studies have reported higher Lp(a) concentrations in women with preeclampsia compared with unaffected pregnancies, suggesting that altered lipid metabolism and pre-existing vascular susceptibility may contribute to placental dysfunction [14–17]. Because Lp(a) concentrations are largely genetically determined and remain relatively stable over time, early pregnancy measurements may reflect baseline maternal vascular susceptibility rather than a secondary consequence of placental disease. However, the potential role of first-trimester Lp(a) as an early susceptibility marker for preeclampsia remains incompletely understood.

Renal involvement is a defining feature of preeclampsia, and uric acid has long been recognized as a biochemical marker associated with severity of the disease [18]. Beyond reflecting impaired renal clearance, uric acid may actively contribute to oxidative stress, inflammation, and endothelial dysfunction [19, 20]. Elevated uric acid levels are typically observed near the time of diagnosis and have been associated with adverse maternal and fetal outcomes [21]. Nevertheless, a few studies have examined whether uric acid trajectories across pregnancy differ between women who develop preeclampsia and those with uncomplicated pregnancies.

Cystatin C has emerged as an additional marker of renal function that may detect subtle changes in glomerular filtration earlier than creatinine and is less influenced by muscle mass. Several studies have reported higher cystatin C concentrations in preeclamptic pregnancies, even before overt clinical manifestations, suggesting early renal involvement in disease pathophysiology. However, longitudinal data on cystatin C trajectories across pregnancy remain limited [22, 23].

Most previous studies evaluating inflammatory, lipid, and renal biomarkers in preeclampsia have relied on cross-sectional designs or measurements obtained close to the clinical onset of disease [8, 17, 18]. Such approaches limit insight into the temporal evolution of biological disturbances preceding preeclampsia. In contrast, longitudinal analyses of repeated biomarker measurements allow assessment of within-individual trajectories and provide a more accurate representation of disease progression.

The aim of this study was therefore twofold: first, to evaluate whether first-trimester lipoprotein(a) concentrations are associated with subsequent development of preeclampsia as a potential marker of baseline vascular susceptibility; and second, to investigate whether renal and inflammatory biomarkers demonstrate distinct longitudinal trajectories across pregnancy in women who subsequently develop preeclampsia compared with those who remain normotensive.

## Materials and methods

### Study design and population

This study was a retrospective analysis of prospectively collected data from a pregnancy cohort followed at Hospital Clínico Universitario “Virgen de la Arrixaca” in Murcia, Spain. Women were enrolled at 11–13 week gestation and followed throughout pregnancy as part of routine clinical care and structured first-trimester risk assessment for preeclampsia. First-trimester screening was performed using the Fetal Medicine Foundation (FMF) algorithm implemented in ViewPoint software, which incorporates maternal characteristics, mean arterial pressure, uterine artery pulsatility index, pregnancy-associated plasma protein-A (PAPP-A), and placental growth factor (PLGF) [24]. Women with a calculated risk of preeclampsia greater than 1:100 were classified as high risk and were offered prophylactic treatment with aspirin 150 mg once daily until 36 weeks of gestation. The present study represents a secondary observational analysis of these prospectively collected data and was not designed to evaluate the effects of any specific therapeutic intervention. Participants with at least one available biomarker measurement in any of the three trimesters and a known pregnancy outcome were included.

Preeclampsia was diagnosed according to American Journal of Obstetrics and Gynecology (AJOG)/American College of Obstetricians and Gynecologists (ACOG) guidelines, defined as new-onset hypertension after 20 weeks of gestation (systolic blood pressure ≥ 140 mmHg and/or diastolic blood pressure ≥ 90 mmHg) along with proteinuria and/or evidence of maternal organ dysfunction [25].

The study was conducted in accordance with the principles of the Declaration of Helsinki. Ethical approval was obtained from the local institutional review board, and all participants provided written informed consent (2016–12-8-HCUVA).

### Biochemical analyses

Maternal blood samples were obtained during routine antenatal visits corresponding to approximately 12, 20, and 32 weeks of gestation. Serum concentrations of interleukin-6, lipoprotein(a), uric acid, cystatin C, and lipid parameters were measured using the standardized laboratory methods according to the manufacturer’s instructions and internal quality-control procedures. Biomarker values were analyzed in original units and log-transformed when required to approximate normal distributions.

Maternal history and outcomes were extracted from medical records, including maternal age, body mass index (BMI), smoking status, and relevant obstetric history. Gestational age was determined based on first-trimester ultrasound dating and incorporated into longitudinal analyses through trimester-specific modeling.

### Statistical analysis

Baseline characteristics were summarized using mean ± standard deviation or median (interquartile range) for continuous variables and counts (percentages) for categorical variables. Group comparisons used Student’s t test or Mann–Whitney U test for continuous variables and χ^2^ or Fisher’s exact test for categorical variables.

Longitudinal changes in inflammatory and renal biomarkers were analyzed using population-averaged longitudinal models accounting for repeated measurements. Models included fixed effects for trimester, preeclampsia status, and their interaction. Adjusted models included maternal age and body mass index.

Given the biological stability of lipoprotein(a), longitudinal modeling was not performed. Instead, first-trimester lipoprotein(a) concentrations were evaluated as a baseline exposure using logistic regression adjusted for maternal age and body mass index. Missing baseline covariates were imputed using cohort median values.

All tests were two-sided with *p* < 0.05 considered statistically significant. Analyses were performed using Python (version 3) with pandas, NumPy, SciPy, and statsmodels.

## Results

### Study population

A total of 119 women were included; 20 (16.8%) developed preeclampsia, while 99 (83.2%) remained normotensive. Overall, 108 of 119 women (90.8%) received prophylactic aspirin during pregnancy according to the first-trimester screening results. Baseline characteristics are shown in Table 1. A history of previous preeclampsia was more frequent among affected women (25.0% vs 7.1%, *p* = 0.045). No other baseline differences reached statistical significance. Among women who developed preeclampsia, 8 cases (40.0%) were classified as early onset (< 34 weeks), 4 (20.0%) as late-onset (≥ 34 weeks), and 8 (40.0%) as term preeclampsia.

**Table 1:** Baseline characteristics

Variable	PE	No PE	*p* value
Maternal age (years)	33.2 ± 4.8	32.9 ± 5.4	0.82
BMI (kg/m^2^)	28.1 (24.2–35.1)	25.6 (22.9–29.1)	0.17
Weight (kg)	76.1 (62.7–84.2)	67.6 (60.5–80.0)	0.36
Height (cm)	161.8 ± 5.7	163.2 ± 5.9	0.46
Asian ethnicity	0/11 (0.0%)	1/98 (1.0%)	1.00
Smoking	1/11 (9.1%)	8/98 (8.2%)	1.00
Multiparity	9/20 (45.0%)	28/98 (28.6%)	0.24
Previous PE	**5/20 (25.0%)**	**7/98 (7.1%)**	**0.045**
Aspirin use	**10 (50.0%)**	**98 (99.0%)**	** < 0.001**

Significant p values appear in bold

Data are presented as mean ± SD, median (IQR), or n/*N* (%), as appropriate

### Baseline biomarker concentrations

First-trimester biomarker concentrations are shown in Table 2. Women who later developed preeclampsia exhibited significantly higher first-trimester lipoprotein(a) concentrations. In adjusted logistic regression analyses, higher lipoprotein(a) was independently associated with an OR of 2.38 (*p* = 0.0001) to develop preeclampsia.

**Table 2:** First-trimester inflammatory, lipid, and renal biomarkers

Biomarker (1st trimester)	PE	No PE	*p* value
Uric acid (mg/dL)	4.25 (3.12–4.48)	3.30 (2.80–3.60)	**0.005**
CRP (mg/L)	1.07 (0.70–1.54)	0.64 (0.32–1.15)	**0.015**
IL-6 (pg/mL)	4.80 (2.21–9.40)	2.71 (1.78–3.44)	**0.017**
Lipoprotein(a) (mg/dL)	45.40 (15.80–85.00)	6.17 (3.75–15.50)	** < 0.001**
Triglycerides (mg/dL)	172.7 (141.8–189.0)	127.7 (102.9–173.1)	0.052
Creatinine (mg/dL)	0.57 ± 0.12	0.62 ± 0.13	0.110
Total cholesterol (mg/dL)	220.0 (208.6–227.7)	205.8 (190.5–240.5)	0.400
Urea (mg/dL)	19.6 (19.2–21.3)	20.0 (18.0–23.0)	0.700
Cystatin C (mg/L)	0.60 (0.55–0.63)	0.59 (0.55–0.66)	0.890
HDL-C (mg/dL)	76.0 ± 23.3	76.3 ± 19.8	0.970

Significant p values appear in bold

Data are presented as mean ± SD, median (IQR), or n/N (%), as appropriate

First-trimester interleukin-6 and C-reactive protein concentrations were higher among women who later developed preeclampsia, although these analyses were unadjusted and descriptive.

### Longitudinal biomarker trajectories

**Fig. 1:**
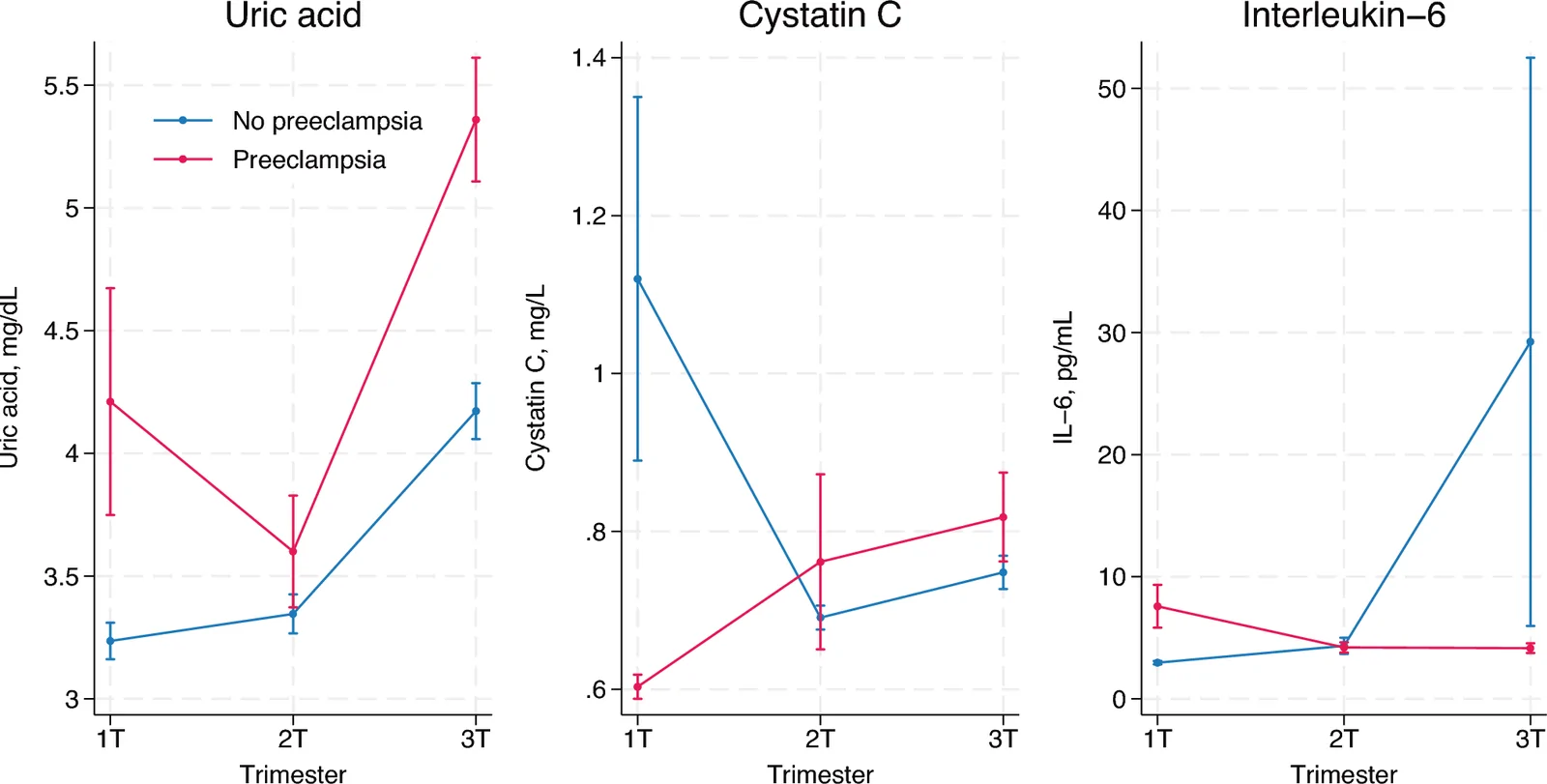
Longitudinal trajectories of inflammatory and renal biomarkers across pregnancy according to preeclampsia. Mean (± standard error) concentrations of (**A**) uric acid, (**B**) cystatin C, and (**C**) interleukin-6 across the first, second, and third trimesters in women who developed preeclampsia and those who remained normotensive. Trajectories were derived from population-averaged longitudinal models adjusted for maternal age and body mass index. Significant trimester-by-preeclampsia interactions were observed for uric acid and cystatin C, but not for interleukin-6

Longitudinal trajectories are shown in Fig. 1 and summarized in Table 3. A total of 286 uric acid and 278 cystatin C measurements were available.

**Table 3:** Longitudinal analysis of biomarker trajectories across pregnancy

Biomarker	Scale	Observations	Subjects	Trimester × PE interaction *p*
Uric acid	Raw	286	116	**0.030**
Cystatin C	Raw	278	118	**0.034**
CRP	Log	252	116	0.071
Creatinine	Raw	301	118	0.104
Urea	Raw	246	117	0.112
IL-6	Log	306	118	0.134
Total cholesterol	Raw	231	112	0.613
Triglycerides	Log	242	114	0.783
HDL cholesterol	Raw	247	113	0.791

Significant p values appear in bold

Population-averaged longitudinal models were used to assess trimester-by-preeclampsia interactions for each biomarker. Models were adjusted for maternal age and body mass index. Biomarkers were analyzed on the raw or log-transformed scale as indicated. The number of observations reflects all available repeated measurements across pregnancy

Adjusted longitudinal models demonstrated significant trimester-by-preeclampsia interactions for uric acid (*p* = 0.03) and cystatin C (*p* = 0.03). Among women who developed preeclampsia, both biomarkers increased progressively across gestation, whereas trajectories in normotensive pregnancies remained relatively stable.

No significant trimester-by-preeclampsia interaction was observed for interleukin-6 or C-reactive protein.

### Exploratory analyses

Among women with preeclampsia, first-trimester lipoprotein(a) concentrations tended to be higher in early onset (< 34 weeks) compared with later-onset disease, although this difference was not statistically significant, likely reflecting the limited sample size.

## Discussion

In this pilot study, we identified distinct baseline and longitudinal biomarker patterns preceding the development of preeclampsia. Specifically, elevated first-trimester Lp(a) concentrations were independently associated with subsequent preeclampsia, supporting the concept of early vascular susceptibility. In addition, renal-related biomarkers, particularly uric acid and cystatin C, demonstrated significantly different longitudinal trajectories across pregnancy in women who developed preeclampsia, whereas inflammatory markers showed early differences with less pronounced longitudinal divergence. Together, these findings support the concept that preeclampsia reflects both an underlying maternal predisposition and progressive biological changes occurring throughout pregnancy rather than a sudden late gestational event.

Several studies have reported higher Lp(a) concentrations in women with established preeclampsia compared with normotensive pregnancies, suggesting a link between altered lipid metabolism and placental vascular pathology [13, 14, 17]. Hubel et al. first described increased Lp(a) levels in preeclamptic pregnancies, proposing a potential role for lipid-related endothelial dysfunction [17]. Subsequent studies by Belo et al. and Enquobahrie et al. extended these observations, demonstrating associations between adverse lipid profiles in pregnancy and preeclampsia risk [13, 14]. However, most of these studies relied on cross-sectional designs or measurements obtained later in gestation, limiting insight into whether elevated Lp(a) represents a cause or consequence of disease.

In the present study, the association between elevated Lp(a) and preeclampsia was evident in the first trimester and remained significant after adjustment for maternal age and body mass index. This temporal relationship supports the interpretation of Lp(a) as a marker of baseline vascular susceptibility, rather than a biomarker that evolves in response to placental dysfunction. Given the largely genetically determined nature of Lp(a) and its relative stability over time [15, 16], our findings are biologically plausible and align with cardiovascular literature linking Lp(a) to endothelial dysfunction and atherothrombotic risk.

Exploratory analyses further suggested higher first-trimester Lp(a) concentrations among women who developed early onset preeclampsia (< 34 weeks) compared with later-onset disease, although the small number of early cases precluded definitive conclusions. This observation is nevertheless consistent with the concept that early onset preeclampsia represents a more placental-driven and vascular phenotype, as opposed to later-onset disease, which may be more strongly influenced by maternal metabolic factors [3–5]. Larger studies are required to determine whether Lp(a) differentially contributes to early versus late-onset preeclampsia.

Renal involvement is a defining feature of preeclampsia, and uric acid has long been recognized as a biochemical marker associated with disease presence and severity [18]. Prior studies have demonstrated that elevated uric acid levels near the time of diagnosis are associated with adverse maternal and fetal outcomes [21], and experimental work suggests that uric acid may actively contribute to oxidative stress, inflammation, and endothelial dysfunction [19, 20]. However, most clinical studies have relied on measurements obtained close to disease onset, limiting understanding of the temporal evolution of renal dysfunction.

In this context, large prospective cohort studies and meta-analyses have shown that elevated uric acid concentrations in early and mid-pregnancy are associated with an increased risk of subsequent preeclampsia, yet these associations have primarily been derived from isolated time-point assessments [26–28]. Our longitudinal findings extend this literature by demonstrating that uric acid levels follow a progressively divergent trajectory across gestation in women who later develop preeclampsia, rather than rising abruptly at the time of clinical diagnosis. This temporal separation is consistent with a time-dependent and dose–response model in which early renal and metabolic maladaptation amplifies over pregnancy, ultimately crossing a clinical threshold. Moreover, this trajectory-based behavior may partly explain the moderate predictive performance observed in prior studies, in which static cutoffs yield high negative but low positive predictive values, and suggests that the clinical value of uric acid lies in its dynamic evolution over time rather than in isolated absolute measurements [28, 29].

The inclusion of cystatin C, a biomarker less affected by muscle mass and more sensitive to subtle alterations in glomerular filtration, further reinforces the evidence for early renal involvement. These findings underscore renal maladaptation as a dynamic and progressive component of preeclampsia pathophysiology.

Importantly, our results are consistent with the body of longitudinal and prospective research assessing cystatin C in preeclampsia. In a nested case–control study within a prospective cohort, Risch et al. demonstrated that elevated first-trimester cystatin C levels, despite normal serum creatinine, were independently associated with subsequent development of preeclampsia, suggesting that impaired glomerular filtration precedes overt clinical disease [30]. Similarly, Thilaganathan et al. reported that higher first-trimester maternal cystatin C concentrations were linked to later preeclampsia, with nearly half of affected women exhibiting values above the 80th percentile of controls, supporting its role as an early marker of renal vulnerability [31]. In contrast, Guo et al. assessed cystatin C longitudinally in later gestation and postpartum, demonstrating pronounced third-trimester elevations in severe preeclampsia and a strong inverse correlation with creatinine clearance, highlighting its value in monitoring disease severity and renal recovery [32].

Our findings integrate and extend these observations by characterizing the trajectory of cystatin C throughout pregnancy, demonstrating that renal dysfunction is not restricted to specific gestational periods but appears to develop progressively well before clinical diagnosis. This temporal pattern supports the concept of preeclampsia as a continuum of renal and endothelial maladaptation rather than an acute, late-gestation disorder, and reinforces the potential value of incorporating longitudinal renal biomarkers into predictive and pathophysiological models of the disease.

Our longitudinal analyses extend these findings by demonstrating that uric acid and cystatin C trajectories diverge progressively across pregnancy in women who develop preeclampsia, preceding clinical diagnosis. This supports a model in which renal and vascular dysfunction evolves gradually during gestation rather than emerging abruptly.

Systemic inflammation has been consistently implicated in preeclampsia pathogenesis [6, 7], and elevated circulating IL-6 concentrations have been reported in women with established disease [8, 9]. In our cohort, inflammatory markers, such as IL-6 and C-reactive protein, were higher early in pregnancy among women who later developed preeclampsia. However, longitudinal modeling did not demonstrate a significant divergence in inflammatory trajectories across gestation. These findings suggest that inflammatory activation may represent an early, relatively stable feature rather than a progressively amplifying process during pregnancy. This interpretation is consistent with the two-stage model of preeclampsia, in which early placental stress initiates systemic changes that subsequently manifest as maternal disease [3–5].

Taken together, our findings support a conceptual framework in which preeclampsia arises from the interaction between baseline maternal susceptibility and progressive gestational maladaptation. Elevated first-trimester Lp(a) may be consistent with an underlying vascular risk profile associated with subsequent development of preeclampsia. In contrast, the longitudinal elevation of renal biomarkers likely captures the evolving maternal response to placental stress as pregnancy advances. This integrated view aligns with contemporary models of preeclampsia pathophysiology and underscores the importance of considering both static and dynamic biological processes.

This study benefits from prospectively collected data, serial biomarker measurements across pregnancy, and longitudinal statistical modeling capable of handling incomplete follow-up.

Limitations include the single-center design and small sample size, particularly for the limited number of preeclampsia cases and early onset preeclampsia, which restricted statistical power and prevented meaningful subgroup analyses. Biomarker sampling was aligned with routine clinical care rather than a predefined research schedule, resulting in some measurements being obtained close to or after the clinical diagnosis of preeclampsia. In addition, adjustment was limited to key maternal characteristics, and residual confounding from unmeasured factors cannot be excluded. As an observational secondary analysis, the study was designed to explore associations and biomarker trajectories rather than establish causal relationships.

As this cohort included women identified as being at increased risk for preeclampsia in the first trimester, a proportion of participants received low-dose aspirin according to contemporaneous clinical practice guidelines. Although the present study was not designed to evaluate treatment effects, aspirin exposure may have influenced the incidence, timing, or severity of preeclampsia, as well as the evolution of certain biomarkers. Furthermore, because of the limited number of cases, separate analyses of early and late-onset preeclampsia were not feasible. As participants were recruited from a cohort identified as being at increased risk for preeclampsia during first-trimester screening, the baseline cardiometabolic profile of the study population may differ from that of an unselected obstetric population. Consequently, the generalizability of these findings to low-risk populations should be interpreted with caution and requires external validation. Larger prospective studies will be required to validate these observations and further explore the influence of aspirin exposure and preeclampsia subtype on biomarker trajectories.

## Conclusions

In this pilot cohort, preeclampsia was associated with higher first-trimester lipoprotein(a) concentrations and distinct longitudinal trajectories of renal biomarkers. Elevated first-trimester lipoprotein(a) concentrations may reflect baseline vascular susceptibility, while divergent longitudinal trajectories of renal biomarkers highlight evolving maternal maladaptation prior to clinical disease onset. These results support a dynamic model of preeclampsia pathophysiology and emphasize the potential value of longitudinal biomarker assessment. Larger, multicenter studies are warranted to validate these observations and to clarify the role of Lp(a) in early versus late-onset disease.

## Supplementary Information

Below is the link to the electronic supplementary material.

Supplementary file1 (PPTX 4990 KB)

## Data Availability

No datasets were generated or analyzed during the current study.
